# Replicability, adaptability and long-term impact of the ‘Healthy Youngsters, Healthy Dads’ program in Newcastle, Australia

**DOI:** 10.1093/heapro/daae095

**Published:** 2024-08-12

**Authors:** Lee M Ashton, Jacqueline A Grounds, Alyce T Barnes, Emma R Pollock, Myles D Young, Stevie-Lee Kennedy, Anna T Rayward, Daniel R Lee, Philip J Morgan

**Affiliations:** Centre for Active Living and Learning, College of Human and Social Futures, School of Education, University of Newcastle, Awabakal Country, Callaghan, New South Wales, 2308, Australia; Active Living and Learning Research Program, Hunter Medical Research Institute (HMRI), Lot 1 Kookaburra Circuit, Awabakal Country, New Lambton Heights, New South Wales 2305, Australia; Centre for Active Living and Learning, College of Human and Social Futures, School of Education, University of Newcastle, Awabakal Country, Callaghan, New South Wales, 2308, Australia; Active Living and Learning Research Program, Hunter Medical Research Institute (HMRI), Lot 1 Kookaburra Circuit, Awabakal Country, New Lambton Heights, New South Wales 2305, Australia; College of Health, Medicine and Wellbeing, School of Medicine and Public Health, The University of Newcastle, Awabakal Country, Newcastle, New South Wales, 2308, Australia; The National Centre of Implementation Science (NCOIS), The University of Newcastle, Awabakal Country, Newcastle, New South Wales, 2308, Australia; Population Health Research Program, Hunter Medical Research Institute, Awabakal Country, New Lambton Heights, New South Wales, 2305, Australia; Hunter New England Population Health, Hunter New England Local Health District, Awabakal Country, Newcastle, New South Wales, 2287, Australia; College of Health, Medicine and Wellbeing, School of Medicine and Public Health, The University of Newcastle, Awabakal Country, Newcastle, New South Wales, 2308, Australia; The National Centre of Implementation Science (NCOIS), The University of Newcastle, Awabakal Country, Newcastle, New South Wales, 2308, Australia; Population Health Research Program, Hunter Medical Research Institute, Awabakal Country, New Lambton Heights, New South Wales, 2305, Australia; Hunter New England Population Health, Hunter New England Local Health District, Awabakal Country, Newcastle, New South Wales, 2287, Australia; Active Living and Learning Research Program, Hunter Medical Research Institute (HMRI), Lot 1 Kookaburra Circuit, Awabakal Country, New Lambton Heights, New South Wales 2305, Australia; College of Engineering, Science and Environment, School of Psychology, University of Newcastle, Awabakal Country, Callaghan, New South Wales, 2308, Australia; Centre for Active Living and Learning, College of Human and Social Futures, School of Education, University of Newcastle, Awabakal Country, Callaghan, New South Wales, 2308, Australia; Active Living and Learning Research Program, Hunter Medical Research Institute (HMRI), Lot 1 Kookaburra Circuit, Awabakal Country, New Lambton Heights, New South Wales 2305, Australia; College of Health, Medicine and Wellbeing, School of Medicine and Public Health, The University of Newcastle, Awabakal Country, Newcastle, New South Wales, 2308, Australia; The National Centre of Implementation Science (NCOIS), The University of Newcastle, Awabakal Country, Newcastle, New South Wales, 2308, Australia; Population Health Research Program, Hunter Medical Research Institute, Awabakal Country, New Lambton Heights, New South Wales, 2305, Australia; Hunter New England Population Health, Hunter New England Local Health District, Awabakal Country, Newcastle, New South Wales, 2287, Australia; Centre for Active Living and Learning, College of Human and Social Futures, School of Education, University of Newcastle, Awabakal Country, Callaghan, New South Wales, 2308, Australia; Active Living and Learning Research Program, Hunter Medical Research Institute (HMRI), Lot 1 Kookaburra Circuit, Awabakal Country, New Lambton Heights, New South Wales 2305, Australia; Centre for Active Living and Learning, College of Human and Social Futures, School of Education, University of Newcastle, Awabakal Country, Callaghan, New South Wales, 2308, Australia; Active Living and Learning Research Program, Hunter Medical Research Institute (HMRI), Lot 1 Kookaburra Circuit, Awabakal Country, New Lambton Heights, New South Wales 2305, Australia

**Keywords:** fathers, preschool children, physical activity, nutrition, parenting

## Abstract

‘Healthy Youngsters, Healthy Dads’ (HYHD) targets fathers to improve the health of their preschool-aged children. In a previous randomized trial, fathers and children experienced meaningful improvements in physical activity and eating behaviours. The next phase is to test the replicability and adaptability of HYHD when delivered in the community by trained facilitators. Fathers/father-figures and children aged 3–5 years were recruited from Newcastle, Australia into a 9-week, non-randomized trial with assessments at baseline, 10 weeks, and 12 months. The primary outcome was achievement of pre-registered targets for recruitment (≥ 96 dyads), attendance (≥ 70%), compliance (completing ≥ 70% of home-based tasks), fidelity (≥ 80% of content delivered as intended) and program satisfaction (≥ 4/5). Secondary outcomes included physical activity, nutrition, screen time and parenting measures. Process targets were surpassed for recruitment (140 fathers, 141 children), attendance (79% for fathers-only workshops, 81% for father–child sessions), compliance (80% of home-tasks completed), fidelity (99% for education, ≥ 97% for practical) and program satisfaction (4.8/5). Mixed effects regression models revealed significant effects in fathers for moderate-to-vigorous physical activity, co-physical activity, dietary intake and parenting practises, which were maintained at 12 months. Significant effects were also established for screen time at 10 weeks only. For children, significant effects were observed for screen time and dietary intake at 10 weeks, while effects on energy-dense, nutrient-poor foods and healthy, nutrient-dense core food intake were maintained at 12 months. Findings demonstrate the replicability and adaptability of HYHD when delivered in the community by local trained facilitators. Further investigation into how to optimally scale-up HYHD is warranted.

**Contribution to Health Promotion**

- Fathers play an important role in promoting physical activity and healthy eating to their young children, but few participate in parenting programs that target these behaviours.
- This study shows the long-term impact of the ‘Healthy Youngsters, Healthy Dads’ program when delivered in the community by trained facilitators. This was demonstrated by robust outcomes for recruitment, attendance, compliance, fidelity and program satisfaction. Additionally, fathers and their preschool-aged children improved physical activity and dietary intake at 10 weeks and 12 months.
- The ability to implement the program within community settings, such as schools, using trained facilitators will support future scale-up of the program.

## INTRODUCTION

There are growing concerns over the health behaviours of young children globally with calls for early intervention to improve physical activity levels and diet quality (World Health Organization, 2016). The World Health Organization recommends at least 180 min of physical activity per day at any intensity, including 60 min per day of moderate-to-vigorous physical activity (MVPA) for children aged 3–4 years. While some studies have shown preschoolers meet physical activity guidelines (Vale *et al*., 2010; Guan *et al*., 2020), evidence from a systematic review of 39 studies found nearly half of preschool-aged children do not engage in sufficient activity (Tucker, 2008). Furthermore, national data in the USA show over half of preschool-aged children to be inactive (Pate *et al*., 2015), while national data in both the UK and Australia show 91% of 2- to 4-year-olds (Scholes, 2016) and 90% of 5-year-olds (Australian Institute of Health and Welfare., 2018b) do not meet daily physical activity recommendations. Simultaneously, large-scale nutrition data show that preschool-aged children internationally have poor diet quality (Angelopoulos *et al*., 2009; Hamner and Moore, 2020), low fruit and vegetable intake (Huybrechts *et al*., 2008; Australian Bureau of Statistics, 2014) and high intakes of energy-dense, nutrient-deficient foods (aka ‘junk foods’) (Pereira-da-Silva *et al*., 2016; Australian Institute of Health and Welfare., 2018a). Early intervention is critical because positive physical activity behaviours and eating habits formed early in life can track into adulthood, reducing risks of developing numerous noncommunicable diseases across the life course (Craigie *et al*., 2011; Telama *et al*., 2014; Hidayat *et al*., 2020).

Concerning health trends are also found in men, with 75% of Australian men living with overweight or obesity (Australian Institute of Health and Welfare., 2023), 76% being insufficiently active and 97% not consuming the recommended fruit and vegetable intake (Australian Institute of Health and Welfare., 2023). Moreover, fatherhood can increase the risk of developing overweight and obesity due to declining physical activity levels, particularly among men with young children (Pot and Keizer, 2016).

Recent systematic reviews have highlighted the importance of including parents in interventions targeting the health behaviours of young children, given the significant role parents play in shaping children’s health (Ash *et al*., 2017; Hnatiuk *et al*., 2019; Nordlund *et al*., 2022). Furthermore, studies are increasingly revealing the unique and powerful impact fathers have in promoting physical activity and healthy eating to their children (Morgan and Young, 2017; Morgan *et al*., 2020; Rahill *et al*., 2020). Despite this, fathers account for just 6% of attendees in parenting programs for childhood obesity treatment and prevention (Morgan *et al*., 2017).

Healthy Youngsters, Healthy Dads (HYHD) was developed to target families’ health behaviours through the father–child relationship. After successful testing in a pilot study (Morgan *et al*., 2021), the HYHD randomized controlled trial (RCT) found young children and fathers who received the intervention significantly improved numerous health behaviours at 10-week and 9-month follow-up when compared with the control group, including increased steps per day (Morgan *et al*., 2022), increased energy intake from core foods and decreased intake of energy-dense, nutrient-poor foods (Ashton *et al*., 2021). Favourable group-by-time effects were seen for children’s fundamental movement skill (FMS) proficiency, and fathers’ physical activity levels, dietary behaviours and several parenting constructs (Ashton *et al*., 2021; Morgan *et al*., 2022), all of which impact child health (Lubans *et al*., 2010; Hardy *et al*., 2012; Jaakkola *et al*., 2016; Davids *et al*., 2017; Morgan and Young, 2017; Young and Morgan, 2017; Baker *et al*., 2019).

There is a paucity of translational research on childhood obesity interventions (Hennessy *et al*., 2019; McCrabb *et al*., 2019) despite an increasingly identified need to accelerate such research (Czajkowski, 2016; Hennessy *et al*., 2019). Using a translational framework can optimize interventions for impact (Guastaferro and Pfammatter, 2023). One such framework is the Sax Institute’s ‘Translational Research Framework’, which is a structured approach to advancing research from initial development to widespread application (Sax Institute, 2016). This framework ensures that effective innovations are thoroughly tested and adapted before being broadly applied, promoting evidence-based improvements in practice and policy. Aligning with the key phases of this framework, HYHD has been tested for feasibility (Morgan *et al*., 2021) and efficacy (Ashton *et al*., 2021; Morgan *et al*., 2022). The next phase is testing the replicability and adaptability of the program. Specifically, this phase looks to explore if the intervention can reproduce the same outcomes under different conditions. Thus, to progress the evidence base for HYHD from intervention testing to intervention replication, the present study explored whether the positive impact of HYHD could still be replicated when delivered in the community by local trained facilitators. The primary aim of this process evaluation was to test if pre-registered process outcome targets could be achieved for: (i) recruitment, (ii) attendance, (iii) compliance, (iv) fidelity and (v) satisfaction. The secondary aims were to test the impact of the program on a range of participant health behaviours (physical activity, dietary intake, screen time) and parenting practises (co-parenting and father self-efficacy) at the end of the intervention (10 weeks post-baseline) and long term at 12-month follow-up.

## METHODS

### Study design

Details on the study methods have been published elsewhere (Ashton *et al*., 2021; Morgan *et al*., 2021, 2022) and previous studies have assessed the impact of the HYHD program under controlled conditions (Ashton *et al*., 2021; Morgan *et al*., 2022). This present study used a non-randomized trial design with assessments at baseline, 10 weeks (post-program) and 12 months. The data were collected from over eight HYHD programs, with four programs in 2019 and four in 2020. Six of the eight programs were delivered in the community across two locations (a local primary school and a local secondary school), while two programs in 2019 were delivered at the University of Newcastle. Originally, the intention was to run all programs in local schools. However, due to the COVID-19 pandemic, schools had restrictions on allowing visitors to use their spaces. Consequently, two programs in 2019 were run at the University of Newcastle, which had sufficient space to account for social distancing rules. The schools that hosted the remaining six programs were large enough to meet space requirements and a COVID-specific safety plan was developed. The study was prospectively registered with the Australian New Zealand Clinical Trials Registry (ACTRN12619001463167) and received institutional ethics approval (H-2017-0381).

### Participants

Participants were recruited in 2019 and 2020 from Newcastle, New South Wales, Australia. Primary recruitment strategies included: a university media release featured in local news outlets (television news, newspaper, radio); social media (Facebook, Instagram, Twitter) and distribution of flyers to local early childcare centres. Of the 2019 study cohort, 71% of families were initially part of the wait-list control group of the previous related RCT (Morgan *et al*., 2022) and were informed about the current study via email. Importantly, these participants had not received any intervention prior to their baseline assessment in the current trial. Fathers were eligible if they were aged ≤ 65 years and a father or father-figure (e.g. biological father, grandfather, uncle, step-father) of a child aged 3–5 years who had not started primary school. From herein, the use of the term father represents both biological fathers and father-figures. Prior to enrolment, fathers with notable pre-existing health conditions (e.g. cardiovascular disease) required doctor’s clearance. All fathers provided written informed consent as well as child assent.

### The Healthy Youngsters, Healthy Dads intervention

#### Program structure and content

Details of the program structure and content delivered in each session have been described elsewhere (Ashton *et al*., 2021; Morgan *et al*., 2021, 2022). Briefly, the 10-session, 9-week program was informed by extensive formative work (Morgan *et al*., 2011, 2014, 2016, 2019, 2020) and supported fathers to optimize their parenting practices in relation to physical activity and nutrition for their preschool-aged children. The intervention comprised three main components, as outlined below:

(1) *Fathers-only workshops (two* 2-*h, delivered via Zoom in 2019 due to the COVID-19 pandemic and delivered face-to-face in 2020 with recorded Zoom option as a backup)—*Fathers learned about evidence-based strategies to improve their own lifestyle behaviours and parenting practices and to improve their children’s physical activity, dietary habits, social-emotional well-being and sports skills.
(2) *Father–child sessions (eight 75-min weekly face-to-face group sessions)*—Each session comprised two components where fathers and children participated together:
  (i) A 20-min educational session based on a weekly theme relating to physical activity and healthy eating. As an engagement strategy, each theme was linked to one of several, animal characters developed for the program, for example, Charlie Chimpanzee (rough and tumble play), and Reg Rhino (vegetables).
  (ii) A 55-min practical session including: rough and tumble play (e.g. sock wrestle), FMS practise (e.g. catching, kicking, throwing games) and health-related fitness (e.g. father–child shuttle carries).
(3) *Home program (~12-min time commitment each week)—*Participants were provided with an activity handbook with a range of activities for fathers and children to complete at home between sessions. The activities included goal setting, FMS practice, physical activity tracking, fathers-only tasks to reinforce positive parenting practices and home challenges that matched each session theme (e.g. nutrition, physical activity).

All sessions were delivered by local facilitators who were trained by the research team. Each year, participants could choose one of four programs that took places at two locations with two Saturday morning timeslots at each location (08:30 am, 10:00 am).

#### Facilitator recruitment and training

A targeted recruitment approach was implemented for facilitators. Specifically, four experienced Physical Education teachers (3 male, 1 female) known to the research team were recruited via email due to their expertise in working with children, communicating with families and delivering practical sessions safely and effectively. All programs in 2019 was delivered by two facilitators, while programs in 2020 were delivered by one facilitator. Facilitators attended a half-day face-to-face training workshop delivered by the lead investigator (P.J.M.) at the University of Newcastle. The workshop focused on program information (e.g. rationale, structure, background) and key tips to deliver informative, engaging and safe education and practical sessions to fathers and preschool-aged children. Facilitators also received access to an online facilitator portal which housed all program resources to support facilitation including; a facilitator manual, PowerPoint slides for all education session content with presentation notes (explaining how to deliver each slide), videos of practical activities and a handbook with information on how to set-up, deliver and modify practical activities.

### Outcomes

Assessments for the eight HYHD programs took place between October 2019 and October 2021 and included assessments at baseline, post-program (10 weeks) and 12 months. All outcomes except pedometer steps/day were collected at all time-points; a decision was made prospectively, prior to participant enrolment, to not collect 12-month data for the secondary outcome pedometer steps/day to reduce participant burden. In the previous efficacy trial (Morgan *et al*., 2022), the longest follow-up data spanned 9 months. However, for the current study, this follow-up period was extended to 12 months. The extension accounts for seasonality effects in nutrition and physical activity outcomes and aims to address a gap in the literature, as there are few family-based health behaviour interventions assessing long-term follow-up (Brown *et al*., 2016). A full description of all measures is outlined in Table 1. Briefly, the primary outcome was the achievement of process outcome targets for recruitment, attendance, compliance, fidelity and program satisfaction. For replicability purposes, the pre-registered targets (see section ‘indicator of success’ in Table 1) were established based on previous process results for the HYHD program (Morgan *et al*., 2021, 2022). Secondary outcomes included a range of physical activity, nutrition, screen time and parenting outcomes. Demographic information included: participant age, fathers’ employment status, Aboriginal and/or Torres Strait Islander identity, education level, relationship status and country of birth. The sex of the enrolled child was also collected, and socioeconomic status was determined using the Australian postal area index of relative socioeconomic advantage and disadvantage (Australian Bureau of Statistics, 2018). All self-reported secondary outcome and demographic data were collected and managed using SurveyMonkey (Survey Monkey Inc, San Mateo, California, USA) for 2019 programs and REDCap electronic data capture tools for 2020 program (Harris *et al*., 2009, 2019).

**Table 1:** Overview of primary and secondary outcome measures

Measure	Description	Timepoint
**Primary outcome—to achieve at least 4 of the 5 process outcome targets**		
1. Recruitment capability	**Measurement tool:** Audit of study enrolment logs. **Indicator of success:** Achievement of recruitment targets for participants (recruitment of 96 families across the eight HYHD programs across 2 years).	Baseline
2. Attendance	**Measurement tool**: Assessed using workshop attendance checklists at the two father-only workshops and the eight father–child sessions. **Metrics/questions**: Reported as % average attendance at the two fathers-only workshops and % attendance for father-and-child sessions on average across the eight weeks. **Completed by:** Program facilitators. **Indicator of success:** At least 70% average attendance at the two fathers-only workshops and the eight father–child sessions.	Post-program (10 weeks)
3. Compliance with home-based program	**Measurement tool:** Assessed by collecting home-program handbooks at the end of the last session and recording the number of home tasks completed by the fathers and their child which were completed each week. **• Completed by:** Member of research team. **Indicator of success:** Successful compliance will be defined as families completing an average of at least 70% of the home-based tasks in the activity handbook.	Post-program (10 weeks)
4. Fidelity of program delivery	**Measurement tool**: Direct observation of program facilitators by a member of the research team during ≥ two sessions per program. A fidelity checklist was developed for the purpose of the study to ensure all content was delivered as intended. To support this and promote accountability, weekly post session reflections were completed by the facilitators. **Metrics/questions**: Observers ‘ticked’ checklist for successful delivery of session content for education and practical activities. For education sessions, this is reported as % of PowerPoint slides successfully delivered in relation to total number of slides intended to be delivered. For practical, this is % of activities successfully delivered in relation to total number of activities intended to be delivered. For the reflections, facilitators were asked to indicate any sessions where they were unable to deliver as intended with option to elaborate where necessary. **Completed by:** Direct observations completed by member of research team, reflections completed by facilitators. **Indicator of success**: Successful delivery of at least 80% of the session content by facilitators, determined by direct observation.	Post-program (10 weeks)
5. Program satisfaction	**Measurement tool:** Assessed using post-program process evaluation online survey developed for the purpose of the study. **Metrics/questions:** Question focused on participants’ satisfaction with overall program. Response was on a 5-point Likert scale where Poor = 1 and Excellent = 5. **Completed by:** Fathers. **Indicator of success:** Defined as a mean score of at least 4 out of 5 for overall program satisfaction question measured via questionnaire using a 5-point Likert scale.	Post-program (10 weeks)
**Secondary outcomes**		
Fathers’ self-report MVPA (min/week)	**Measurement tool:** Adapted version of the Godin Leisure Time Exercise Questionnaire (Godin and Shephard, 1985), validated for use in healthy adults (Amireault and Godin, 2015). **Metrics/questions:** Fathers reported average weekly bouts of moderate and vigorous physical activity and average bout length (Plotnikoff *et al*., 2006). Values in each category were multiplied and summed to give an overall measure of weekly MVPA. **Completed by:** Fathers.	Baseline, 10 weeks, 12 months
Father–child co-physical activity (days/week)	**Measurement tool:** Two items adapted from the validated Youth Media Campaign Longitudinal Survey (Lee *et al*., 2010), which has been used in previous research involving fathers and their children (Lloyd *et al*., 2015; Morgan *et al*., 2019). **Metrics/questions:** Fathers reported on days per week they were physically active with their child one-on-one and with one or more family member. **Completed by:** Fathers.	Baseline, 10 weeks, 12 months
Fathers’ and child’s screen time	**Measurement tool:** Adapted version of the Adolescent Sedentary Activity Questionnaire (Hardy *et al*., 2007). **Metrics/questions:** Fathers reported the total time they spent sitting using screens (of any kind) for anything outside of work on each day in the previous week. Fathers also answered these questions on behalf of their participating children. This adapted measure has shown good sensitivity to change in previous behaviour change research (Morgan *et al*., 2019). **Completed by:** Fathers.	Baseline, 10 weeks, 12 months
Fathers’ self-efficacy	**Measurement tool:** Positive Engagement and Direct Care subscales of the Fathering Self Efficacy scale (Sevigny *et al*., 2016) **Metrics/questions:** Questions focused on fathers’ efficacy in engagement with child (e.g. *I can always think of fun things to do with my child)* and direct care (e.g. *I can provide the daily care for my child needs*). Total of 16 items and responses were on a 9-point scale ranging from 1 = completely disagree to 9 = completed agree. **Internal consistency on current sample:** α = 0.81. **Completed by:** Fathers.	Baseline, 10 weeks, 12 months
Co-parenting (mothers and fathers)	**Measurement tool:** Feinberg’s Co-parenting scale (Feinberg *et al*., 2012). **Metrics/questions:** 12 items relating to how fathers and mothers/partners work together as parents (e.g. *my partner and I have the same goals for our child*) with responses on 6-point scale from 0 = not true of us to 6 = very true of us. Plus, two items relating to conflict (e.g. *Do one or both of you say cruel or hurtful things to each other in front of your child?)* with response on 6-point scale ranging from 0 = Never to 6 = very often, several times a day. **Internal consistency on current sample:** α = 0.84 (working together items) and α = 0.90 (conflict items). **Completed by:** Fathers.	Baseline, 10 weeks, 12 months
Dietary intake	**Measurement tool:** Adapted version of the Many Rivers Short Food Frequency Questionnaire (MRSFFQ) (Gwynn *et al*., 2011). **Metrics/questions:** Intake is assessed on ordinal scales for 10 dietary outcomes. Fathers responded about themselves and on behalf of their children. Intake of healthy, nutrient-dense core food items of fruit, vegetables and water was scored on a scale 0–4 for fruit and 0–5 for vegetables and water with higher numbers corresponding to higher numbers of serves a day. Energy-dense, nutrient-poor food items of hot chips, salty snacks, confectionary, sweet foods, fast food, fruit juice and sweetened beverages were reverse scored on a scale of 0–6 with higher numbers corresponding to lower numbers of serves per week. Composite variables were then formed through summing across healthy, nutrient-dense core foods, Energy-dense, nutrient-poor food and all items. **Internal consistency on current sample:** α = 0.66 (fathers) and α = 0.60 (father proxy for child). **Completed by:** Fathers.	Baseline, 10 weeks, 12 months
Average steps/day^*^	**Measurement tool:** One week of pedometry using YAMAX SW200 (Yamax Corporation, Kumamoto City, Japan) pedometers (average steps/day used for analysis where at least 4 days are recorded, including at least 1 weekend day) (Silcott *et al*., 2011). Previous research has established convergent validity of the YAMAX SW200 pedometer (*r* = 0.73) when compared with MTI 7164 Actigraph Accelerometer in preschool-aged children (Cardon and De Bourdeaudhuij, 2007). In addition, the YAMAX SW200 pedometer has demonstrated moderate test-retest reliability (ICC = 0.71) and validity when compared with gold standard ActivPAL accelerometer (ICC = 0.96) in adults (Kooiman *et al*., 2015). These findings, along with low relative cost and participant burden supports the use of this tool over other device-based measures. **Completed by:** Fathers and children	Baseline, 10 weeks

^*^ *Collected in 2020 sample only*.

#### Data analyses

For baseline characteristics, data checking and exploratory data analysis involved generation of tables and frequency distributions for categorical variables and summary statistics for continuous variables stratified by year of program participation.

To test the primary aim: if pre-registered process outcome targets could be achieved for: recruitment, attendance, compliance, fidelity and satisfaction; descriptive analyses (i.e. percentage and frequency counts) were conducted.

To test the secondary aim: the impact of the program at the end of the intervention (10 weeks post-baseline) and long-term at 12-month follow-up; mixed effects regression models were used. Mixed effects linear regression, adjusted for cohort year and previous involvement in the RCT (these were the 71% of the 2019 cohort who were part of the wait-list control group of the previous related RCT), were used to compare fathers’ self-efficacy score, co-parenting score and all three dietary intake variables (father and child) at each of the applicable follow-up times. The outcomes of MVPA and weekly average screen time (father and child) were count data and distributions of these outcomes exhibited right skew and overdispersion, with variance significantly larger than the mean. Linear regressions were initially considered, but inspection of residual plots revealed multiple outliers. A Poisson regression model was unsuitable due to overdispersion and therefore a mixed effects negative binomial regression was implemented, adjusted for cohort year and involvement in the RCT to compare these outcomes at each of the applicable follow-up times. For the outcome, average steps/day (father and child) model selection was influenced by outliers, especially in the child data. Therefore, a mixed effects gamma regression, with no adjustment, was used to compare this outcome at each of the applicable follow-up times as this accommodates for high outliers, and its residual plots supported a better fit than linear regression. Other studies measuring physical activity outcomes have used similar distributions (Vandelanotte *et al*., 2021). Random individual-level intercepts were included in all models to account for repeated measurements on the same individual. Correlation between individuals who undertook the program in the same location with the same facilitator was also investigated through nested random intercept models. Correlation was retained only in models for father outcomes of average screen time and the dietary intake variables. In all other instances, the correlation was found to be negligible and removed from the model. Model estimates (rate ratio [RR], or average difference) with 95% confidence intervals (CIs) and *p*-values are presented. For all models, statistical significance is assessed at the 5% level. Mixed effects hurdle gamma regression models, with random individual-level intercepts, were used to investigate outcomes of father–child co-physical activity (solo and family) and number of nights dinner consumed in front of TV (father and child). These models separate the outcome into a binary and gamma component. For the binary component, a 1 is given when the outcome has a value of zero and a 0 is given for non-zero values of the outcome. The binary component is then modelled via a mixed effects logistic regression with an odds ratio (OR) with 95% confidence interval (CI) and *p*-value presented. Non-zero outcome values are then modelled via a mixed effects gamma regression with a rate ratio (RR) with 95% CI and *p*-value presented. Missing data were handled using maximum likelihood estimations from the mixed modelling framework (Enders, 2022).

Sensitivity analyses were carried out with the regression models described above repeated with the fixed effect for previous involvement in the RCT removed. Model estimates were then compared to those from the main analyses. Note: sensitivity analysis was not conducted in the models for father and child average steps/day because data for this outcome was only collected in 2020 participants and none of these participants were previously involved in the RCT. All statistical analyses were programmed using SAS v9.4 (SAS Institute, Cary, North Carolina, USA).

## RESULTS

### Primary outcome—to achieve at least 4 of the 5 process outcome targets

The HYHD program was successfully delivered in community settings by local trained facilitators in eight programs over a 2-year period. This was confirmed by achieving all pre-registered, process outcome targets as outlined below:

#### Recruitment capability (Target: 96 families across the 8 HYHD programs across 2 years)

**Fig. 1:**
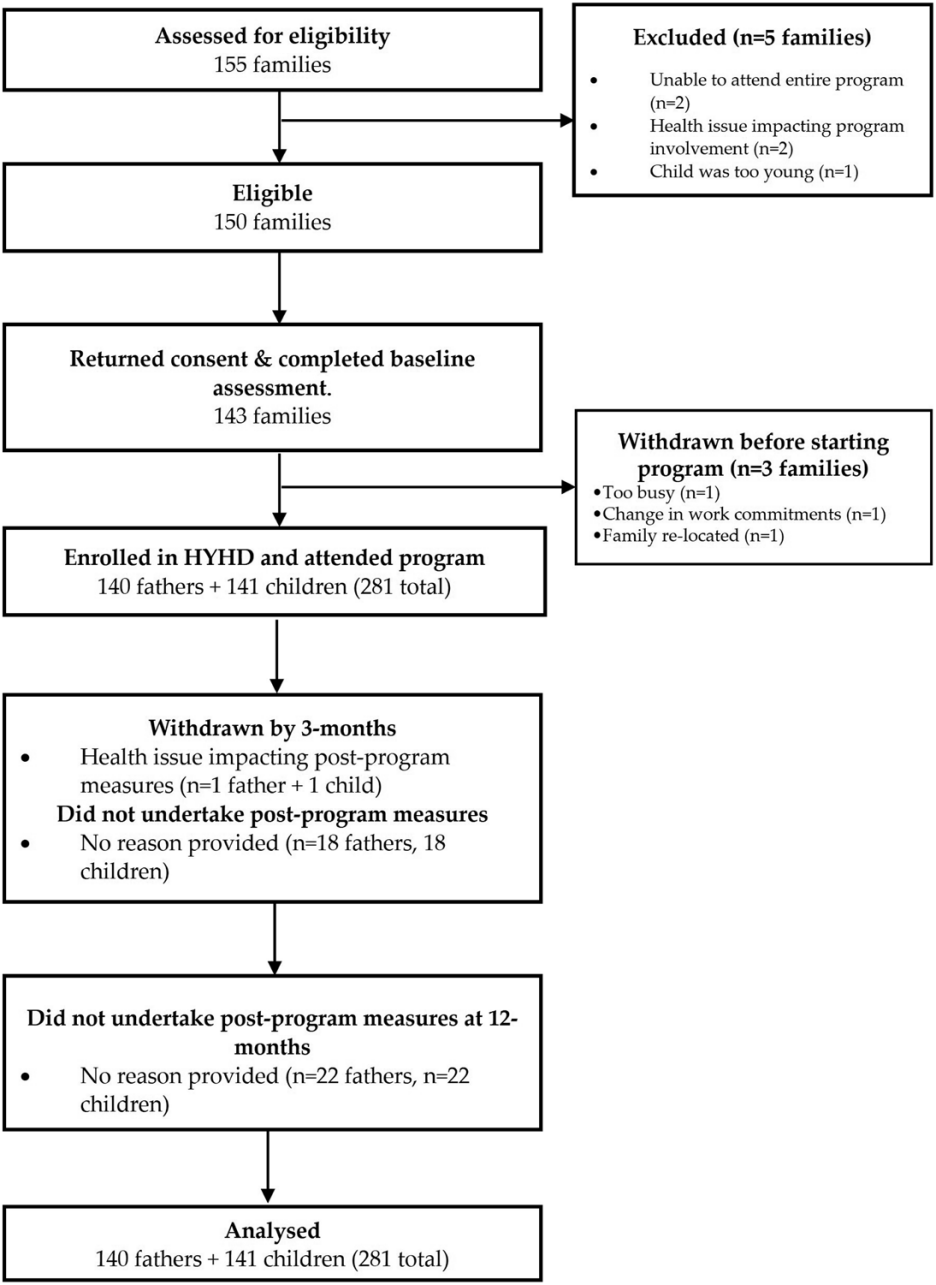
Participant flow through the HYHD program.

Over the 2 years, 155 families expressed interest in the HYHD program. Of these, 97% (*n* = 150 families) met the eligibility criteria (Figure 1). Of those eligible, 143 families provided consent and completed baseline assessments. From this, 140 families (140 fathers + 141 children) enrolled in the program and attended at least one session, which exceeded the recruitment target of 96 families. Of the 2019 study cohort, *n* = 46 (71%) families were initially part of the wait-list control group of the previous related RCT. It took 42 days to recruit the remaining 19 families, which equates to approximately 0.45 families recruited each day. In 2020, recruitment was more efficient, with 75 families recruited in 46 days, equating to 1.6 families recruited per day.

On average, fathers were 37.8 ± 5.3 years of age. Most were living in areas of medium socio-economic status (*n* = 67, 55%), while 73% had a university degree or higher university degree, 90% were working in full-time employment, 84% were married, 84% born in Australia and 99% did not identify as Aboriginal or Torres Strait Islander. For children, the average age was 4.1 ± 1.1 years and 60% were male. Demographic characteristics of study participants split by year of enrolment and the total sample are shown in Table 2. A total of 86% (*n* = 121 families) were retained at the end of the program and undertook post-program measures, while 84% (*n* = 117 families) undertook 12-month follow-up measures.

**Table 2:** Demographic characteristics of the sample (*n* = 281)

		Program year		
Variable	Category	2019 (*n* = 65 fathers, *n* = 65 children)	2020 (*n* = 75 fathers, *n* = 76 children)	Total sample (*n* = 140 fathers, *n* = 141 children)
Age of father at baseline (years)	Mean (SD)	37.9 (4.8)	37.7 (5.6)	37.8 (5.3)
Age of child at baseline (years)	Mean (SD)	3.9 (0.5)	4.3 (1.4)	4.1 (1.1)
SEIFA decile category^a^	Low (1–3)	8 (17.0%)	3 (4.0%)	11 (9.0%)
	Medium (4–7)	26 (55.3%)	41 (54.7%)	67 (54.9%)
	High (8–10)	13 (27.7%)	31 (41.3%)	44 (36.1%)
Sex of enrolled child	Male	45 (69%)	40 (53%)	85 (60%)
Aboriginal or Torres Strait Islander status of father	Do not identify as Aboriginal or Torres Strait Islander	63 (98%)	75 (100.0%)	138 (99.3%)
Australian born	Yes	57 (89%)	60 (80%)	117 (84%)
Relationship status	In a relationship	3 (4.7%)	–	3 (2.2%)
	Living with a partner	9 (14%)	9 (12%)	18 (13%)
	Married	51 (80%)	66 (88%)	117 (84%)
	Separated	1 (1.6%)	–	1 (0.7%)
Highest level of qualification	School certificate (year 10 or equivalent)	3 (4.7%)	–	3 (2.2%)
	Higher school certificate (year 12 or equivalent)	4 (6.3%)	2 (2.7%)	6 (4.3%)
	Trade/Apprentice	6 (9.4%)	3 (4.0%)	9 (6.5%)
	Certificate/Diploma	8 (13%)	12 (16%)	20 (14%)
	University degree	26 (41%)	32 (43%)	58 (42%)
	Higher university degree	17 (27%)	26 (35%)	43 (31%)
Employment status	Full-time paid	59 (92%)	66 (88%)	125 (90%)

^a^Socio-economic status by population decile for SEIFA Index of Relative Socio-economic Advantage and Disadvantage 2016.

#### Attendance (Target: at least 70% average attendance at the two fathers-only workshops and the eight father–child sessions)

Average attendance for the two fathers-only workshops was 78.6%, while average attendance for the eight-weekly father–child sessions was 80.9%. Both exceeded the pre-registered target of ≥ 70%.

#### Compliance with home tasks (Target: families complete on average at least 70% of the home-based tasks in the activity handbook)

Families were encouraged to complete weekly tasks as presented in an activity handbook with a choice of activities for fathers and children to complete at home between sessions. Overall, families completed on average 80% of home-based tasks.

#### Fidelity (Target: successful delivery of ≥ 80% of the session content by facilitators during the fathers-only workshops and the father and child education and practical sessions on average)

Across the 20 sessions observed (10 sessions per year), the feasibility benchmark (≥ 85%) was exceeded with almost all content delivered as intended by the facilitators, with successful delivery of 99% of the education session content delivered. For the practical sessions, 97% of RTP activities, 100% of FMS activities and 100% fitness activities were delivered.

Findings from the direct observation aligned directly with the facilitator’s reflections, with all facilitators indicating that the education content was delivered as intended, while some practical activities were slightly modified on some weeks due to adverse weather (e.g. fitness activities were shorter in duration during sessions with extreme heat) or when the ground was too wet (e.g. ground-based R&T play activities were replaced with standing activities)

#### Satisfaction (Target: mean score of ≥ 4 out of 5 for overall program satisfaction question measured via questionnaire using a 5-point Likert scale)

A list of all acceptability and satisfaction findings is provided in Table 3, while a breakdown of responses is provided in the form of a stacked bar diagram in Supplementary File S1 (Figures 1 and 2). Briefly, fathers considered the overall quality of the program to be high. On a scale of 1 (poor) to 5 (excellent), the mean [standard deviation (SD)] overall program satisfaction score was 4.8 (0.5), which exceeded the pre-registered target (≥4 out of 5). In addition, fathers’ perceptions of the program facilitators were high with a mean (SD) satisfaction score of 4.8 (0.4). Average scores were lower regarding the broader acceptability to wives/partners and the enjoyment, worthwhileness and added value of the fathers-only workshops.

**Table 3:** Acceptability and satisfaction findings as reported by fathers (*n* = 121)

Construct	Questions asked^a^	Mean	SD
Satisfaction	Overall, I enjoyed the Healthy Youngsters, Healthy Dads program.	4.6	0.6
	I would recommend the program to my friends.	4.7	0.6
	The benefits of the program outweighed any disruption to our normal family routine.	4.5	0.7
	The fathers-only workshops were enjoyable	3.9	0.9
	Overall program satisfaction^b^	4.8	0.5
Quality of the fathers-only workshops	The fathers-only workshops were a worthwhile commitment	4.0	0.9
	The fathers-only workshops added value to the rest of the program	4.1	0.9
Broader acceptability	I included my wife/partner in the program by discussing/sharing the session content	4.0	0.7
	My wife/partner and/or other family member/s participated in the weekly home activities with my youngsters and I	3.4	0.9
Quality of facilitators	Overall rating of facilitators^b^	4.8	0.4

^a^1 = strongly disagree; 2 = disagree; 3 = neutral; 4 = agree; 5 = strongly agree.

^b^1 = poor; 2 = fair; 3 = average; 4 = good; 5 = excellent.

### Secondary outcomes

#### Summary tables for all secondary outcomes

Frequency and summary tables corresponding to the physical activity, screen time, parenting outcomes and dietary intake of fathers and children, stratified by timepoint, are shown in Supplementary File S1, Tables 1–4.

#### Regression models—Father outcomes

Model estimates obtained from the mixed effects regression models are shown in Table 4. Relative to baseline, fathers significantly increased weekly minutes of MVPA by 30% (95% CI: 14%, 49%, *p* < 0.001) at 10 weeks and by 37% at 12-month follow-up (95% CI: 14%, 57%, *p* < 0.001). Neither previous involvement in RCT nor year the participant participated in the program demonstrated a statistically significant effect on weekly minutes of MVPA. For fathers’ average daily steps measured with pedometer, there was no statistically significant impact of program participation at 10 weeks post-program.

**Table 4:** Model estimates with 95% CI and *p*-value for father (*n* = 140) and child outcomes (*n* = 141)

Outcome	Model distribution	Type of estimate	10 weeks change from baseline		12 months change from baseline	
			Estimate (95% CI)	*p*-value	Estimate (95% CI)	*p*-value
**Fathers**						
** Physical activity**						
MVPA (mins/week)	Negative binomial	Rate ratio	1.30 (1.14, 1.49)	**0.00**	1.37 (1.19, 1.57)	**0.00**
Average steps/day^a^	Gamma	Rate ratio	0.99 (0.92, 1.06)	0.72	–	**–**
**Co-physical activity**						
With child only (days/week)	Binary	Odd ratio	8.74 (3.09, 24.69)	**0.00**	4.07 (1.69, 9.82)	**0.00**
	Gamma	Rate ratio	1.27 (1.12, 1.43)	**0.00**	1.05 (0.92, 1.18)	0.48
With child and family (days/week)	Binary	Odd ratio	4.29 (1.43, 12.89)	**0.01**	12.68 (2.79, 57.62)	**0.00**
	Gamma	Rate ratio	1.27 (1.13, 1.44)	**0.00**	1.11 (0.98, 1.25)	0.31
**Screen time**						
Weekly average minutes of screen time	Negative binomial	Rate ratio	0.86 (0.78, 0.94)	**0.00**	0.93 (0.85, 1.01)	0.10
**Father self-efficacy**	Gaussian	Average change	5.66 (3.05, 8.27)	**0.00**	3.52 (0.90, 6.14)	**0.01**
**Co-parenting score**	Gaussian	Average change	1.19 (0.20, 2.18)	**0.02**	1.41 (0.37, 2.44)	**0.01**
**Dietary intake**						
Energy-dense, nutrient-poor foods	Gaussian	Average change	1.61 (1.03, 2.19)	**0.00**	0.84 (0.26, 1.43)	**0.00**
Healthy, nutrient-dense core foods	Gaussian	Average change	0.99 (0.67, 1.31)	**0.00**	0.81 (0.49, 1.13)	**0.00**
Total dietary intake	Gaussian	Average change	2.60 (1.93, 3.28)	**0.00**	1.66 (0.98, 2.34)	**0.00**
**Nights eating dinner in front of TV**	Binary	Odds ratio	3.82 (1.61, 9.06)	**0.00**	1.66 (0.98, 2.38)	**0.00**
	Gamma	Rate ratio	0.78 (0.65, 0.94)	**0.01**	1.00 (0.85, 1.18)	0.98
**Preschool-aged children**						
**Physical activity**						
Average steps/day^a^	Gamma	Rate ratio	0.99 (0.93, 1.060)	0.77	–	**–**
**Screen time**						
Weekly average minutes of screen time	Negative binomial	Rate ratio	0.76 (0.76, 0.82)	**0.00**	1.03 (0.95, 1.12)	0.47
**Dietary intake**						
Energy-dense, nutrient-poor foods	Gaussian	Average change	0.84 (0.32, 1.35)	**0.00**	0.03 (−0.49, 0.54)	0.92
Healthy, nutrient-dense core foods	Gaussian	Average change	0.97 (0.65, 1.28)	**0.00**	1.01 (0.69, 1.33)	**0.00**
Total dietary intake	Gaussian	Average change	1.81 (1.22, 2.39)	**0.00**	1.03 (0.43, 1.62)	**0.00**
**Nights eating dinner in front of TV**	Binary	Odds ratio	3.82 (1.61, 9.06)	**0.00**	0.66 (0.30, 1.45)	0.30
	Gamma	Rate ratio	0.78 (0.65, 0.94)	**0.01**	1.00 (0.85, 1.18)	0.98

Bold denotes a significant difference.

^*^Adjusted for previous involvement in RCT as a wait-list control group and cohort year.

^a^Only collected in 2020 participants and at post-intervention. These models are not adjusted for any confounders as only 2020 participants had available data with none of these participants previously involved in the program.

MVPA; moderate-to-vigorous physical activity.

For co-physical activity, fathers were 8.74 (95% CI: 3.09, 24.69, *p* < 0.001) times more likely and 4.07 (95% CI: 1.69, 9.82, *p* < 0.001) times more likely than at baseline to have co-physical activity one-on-one with their child on at least 1 day per week at 10-week and 12-month follow-up, respectively. Similarly, fathers were 4.29 (95% CI: 1.43, 12.89, *p* < 0.001) times more likely and 12.68 (95% CI: 2.79, 57.62, *p* < 0.001) times more likely than at baseline to have co-physical activity with their child and other family members on at least 1 day per week at 10-week and 12-month follow-up, respectively. Regarding the effect on the number of days participating in co-physical activity with the child (gamma models), fathers significantly increased days/week by 27% (95% CI: 12%, 43%, *p* < 0.001) at 10 weeks when compared with baseline but this effect was lost at 12 months. Likewise, fathers significantly increased the days/week of co-physical activity with child and other family members by 27% (95% CI: 13%, 44%, *p* < 0.001) at 10 weeks when compared with baseline but this effect was lost at 12 months. No statistically significant effect was observed for previous program involvement nor cohort year.

Relative to baseline, fathers significantly reduced minutes/week of screen time by 14% (95% CI: 6%, 22%, *p* < 0.001) at 10 weeks. However, this effect was not sustained at 12-month follow-up. No statistically significant effect was observed for previous program exposure nor cohort year.

For fathers’ self-efficacy score, an average 5.66 (95% CI: 3.05, 8.27, *p* < 0.001) point increase was observed at post-program versus baseline with an on average 3.52 (95% CI: 0.90, 6.14, *p* = 0.01) point increase observed at 12-month follow-up versus baseline. For co-parenting score, an on average 1.19 (95% CI: 0.20, 2.18, *p* = 0.02) point increase was observed at 10 weeks versus baseline and an on average 1.41 (95% CI: 0.37, 2.44, *p* = 0.01) point increase observed at 12-month follow-up versus baseline. No statistically significant effects were observed from previous program exposure nor cohort year.

Dietary outcomes were assessed using composite scores and need to be interpreted cautiously with clinical relevance taken into consideration. Models investigated the impact of program participation on energy-dense nutrient-poor foods, healthy nutrient-dense core foods and total dietary intake. For all models, a positive estimate indicates an improvement in dietary intake. A statistically significant increase in intake score was observed for all three dietary outcomes at 10 weeks (all *p* < 0.001) and at 12 months (all *p* < 0.001) relative to baseline. No statistically significant effects were observed for previous program involvement nor cohort year for both the energy-dense, nutrient-poor foods and healthy, nutrient-dense core foods subscales. However, both predictors showed a statistically significant effect for the total dietary intake outcome with previous involvement and participation in 2020 both showing a statistically significant decrease in total dietary intake score.

Investigations on the impact of the program on fathers’ eating dinner in front of the TV on at least one night of the week (binary models) found a statistically significant impact at 10 weeks (OR: 3.82, 95% CI: 1.61, 9.06, *p* < 0.001) when compared with baseline. However, this was not observed at the 12-month follow-up. Regarding the effect on the number of days dinner was eaten in front of TV (gamma models), fathers significantly reduced days/week by 22% (95% CI: 6%, 35%, *p* < 0.001) at 10 weeks when compared with baseline but this effect was lost at 12 months. No statistically significant effects were observed for previous program involvement nor cohort year.

#### Regression models—Child outcomes

Model estimates obtained from the mixed effects regression models are shown in Table 4. For child average daily steps measured with pedometer, there was no statistically significant impact of program participation on children at 10 weeks post-program.

Relative to baseline, children significantly reduced weekly minutes of screen time by 24% (95% CI: 18%, 30%, *p* < 0.001) at 10 weeks. However, this effect was not sustained at 12-month follow-up. No statistically significant effects were observed for previous program involvement nor cohort year.

A statistically significant increase in intake score was observed for all three dietary outcomes at 10 weeks relative to baseline (all *p* < 0.001), while a statistically significant increase in healthy, nutrient-dense core foods (*p* < 0.001) and total dietary intake (*p* < 0.001) was observed at 12-month follow-up. No effects were observed at 12 months for energy-dense, nutrient-poor foods in children. No statistically significant effects were observed for previous program involvement nor cohort year for both the energy-dense, nutrient-poor foods and total dietary intake. However, previous program involvement in RCT showed a statistically significant increase in healthy, nutrient-dense core foods intake score. Cohort year did not show a statistically significant effect for healthy, nutrient-dense core foods intake score.

Investigations on the impact of the program on children eating dinner in front of the TV on at least one night of the week (binary models) found a statistically significant impact at 10 weeks (OR: 3.82, 95% CI: 1.61, 9.06, *p* < 0.001) when compared to baseline but was not observed at the 12-month follow-up. Regarding the effect on the number of days dinner was eaten in front of TV (gamma models), children significantly reduced days/week by 22% (95% CI: 6%, 35%, *p* = 0.01) at 10 weeks when compared with baseline but this effect was lost at 12 months. No statistically significant effects were observed for previous program involvement nor cohort year.

#### Sensitivity analyses

The sensitivity of the mixed effects regression models to the presence of a confounder for previous involvement in the program as wait-list control group was examined. Previous involvement in the program only impacted the 2019 study cohort. For these sensitivity analyses, regression models were the same as main analysis but with fixed effect for previous involvement in the RCT removed. A comparison of model estimates, 95% CI and *p*-values from models with (main analysis) and without (sensitivity analysis) the confounder for previous participation is shown in Supplementary File S1, Table 5 for father outcomes and Table 6 for child outcomes. For outcomes related to fathers, nil to very small changes were observed in model estimates related to the effect observed 10 weeks post-program versus baseline and 12-month follow-up versus baseline. Some changes in interpretation were observed for model estimates related to year of program participation (cohort year). The estimates for cohort year for outcomes of father self-efficacy score and eating dinner in front of TV (binary model) changed from being statistically insignificant to statistically significant, with both having an associated increase in the value of the estimate. The estimates for cohort year for outcomes of energy-dense, nutrient-poor food intake and total dietary intake changed from being statistically significant to statistically insignificant with the value of the estimate moved closer to zero for both outcomes. For outcomes related to children, nil to very small changes were observed in model estimates, with no changes in interpretation found.

## DISCUSSION

This study aligned with the replicability and adaptability phase of the Australian Sax Institute’s Translational Research Framework (Sax Institute, 2016). Of note, HYHD reproduced the same outcomes when delivered in the community by local trained facilitators. The primary aim was assessed by meeting pre-registered targets for recruitment, attendance, compliance, fidelity and satisfaction, while secondary outcomes assessed change in health behaviours and parenting practices at 10 weeks post-intervention and 12 months. Overall, findings were promising and broadly consistent with the HYHD RCT (Ashton *et al*., 2021; Morgan *et al*., 2022), as confirmed by comparable process outcomes (Table 5). In addition, the current study found significant intervention effects for fathers’ physical activity, screen time, dietary intake and positive parenting practices. For children, intervention effects were observed for screen time and dietary intake. Encouragingly, many of these effects were sustained at 12 months for fathers and children which enhanced the external validity of these findings.

**Table 5:** Comparable process outcomes for the current study vs HYHD RCT

Comparable indicator	Current study delivered in the community by trained facilitators	HYHD RCT in controlled settings (Morgan *et al*., 2022)
Recruitment capability	17.5 families recruited per program.	17.8 families recruited per program.
Attendance	79% fathers-only workshops and 81% for weekly father–child sessions.	96% fathers-only workshops and 86% for weekly father–child sessions.
Fidelity	Facilitator delivered 97% of RTP activities, 100% of FMS activities and 100% fitness activities as intended.	Facilitator delivered 95% of RTP activities, 93% of FMS activities and 90% fitness activities as intended.
Overall program satisfaction	Mean 4.8 ± 0.5	Mean 4.8 ± 0.5
Satisfaction with facilitators	Mean 4.8 ± 0.4	Mean 4.9 ± 0.5

This study achieved its primary goal which was to achieve at least four of the five pre-registered process outcome targets for recruitment, attendance, compliance, fidelity and satisfaction. In terms of recruitment, enrolling 140 fathers and 141 preschool-aged children from the community is promising, especially as fathers have previously been difficult to engage in behaviour change interventions and parenting programs (Lundahl *et al*., 2008; Morgan *et al*., 2017). The recruitment success may be attributable to our targeted strategies, which focused on unique paternal motivators (e.g. the opportunity for co-physical activity and quality one-on-one time with their child) and emphasized the father-only nature of the program, as these have been shown to be important for men’s participation in health research (Young *et al*., 2012; Morgan *et al*., 2016). Of the 2019 study cohort, *n* = 46 (71%) families were initially part of the wait-list control group of the previous related RCT and it took 42 days to recruit the remaining 19 families in 2019. Therefore, a greater recruitment period and increase in recruitment efforts would have been required had we not obtained participants from the previous RCT.

The community-trained facilitators were provided with a robust training workshop and access to detailed program resources to support their facilitation. These factors are likely to have led to both the delivery of the program as intended with high-fidelity outcomes and also the favourable process outcomes and improvements in health behaviours among the fathers and children in this current study since studies indicate that aiding individuals responsible for implementing physical activity initiatives for children through informal education and professional development opportunities (e.g. training and resources), can enhance the execution of programs and children’s engagement in physical activity (Casey *et al*., 2014; Koh *et al*., 2016; Morgan *et al*., 2023). To enable future dissemination and achieve scale-up, a larger pool of trained facilitators is required.

The average attendance rates (average of 79% for fathers only and 81% for father–child sessions) are encouraging, especially when compared with similar programs targeting parents and preschool-aged children. For example, in the MEND (Mind, Exercise, Nutrition … Do It!) program for 2- to 4-year-olds, average attendance was 82% (Skouteris *et al*., 2016), while in the KAN-DO study, 46% of parents attended the required group class (Østbye *et al*., 2012) and in the ‘Hip Hop for Health’ program, 38% of parents attended at least one of the six classes offered (Fitzgibbon *et al*., 2013). It is likely that the high attendance rates in the current study is linked to high program satisfaction ratings for the program (mean: 4.8 out of 5). This, coupled with high home-program compliance, may further explain the positive findings established within this current study.

At post-intervention, fathers increased weekly minutes of MVPA by 30% compared to baseline and this effect was sustained at 12 months. This is an important finding given that 74% of Australian adult males (18–64 years) failed to meet national physical activity guidelines (Australian Bureau of Statistics, 2022) and that men’s physical activity typically decreases during fatherhood (Pot and Keizer, 2016). Furthermore, in a review of physical activity and nutrition interventions for adult men, less than half of the studies demonstrated significant increases in physical activity outcomes (George *et al*., 2012). This further substantiates the positive findings among fathers within the current study and could be attributed to the targeted reciprocal reinforcement (e.g. father and children encourage each other be active), and co-physical activity among the father and child. Especially given that findings confirmed that fathers were 8.7 times more likely to engage in one-on-one co-physical activity with their child on at least 1 day per week at post-program compared to baseline.

Despite the positive self-report findings, objectively measured physical activity using pedometers (steps/day) found no change in either fathers or children at post-program. This could be attributable to non-compliance of wearing pedometers, with greater support provided to comply during the RCT. There were several reasons provided for non-wear time with the most common relating to forgetting to wear, sickness, injury, removal for swimming and refusal to wear. Additionally, pedometers can produce variability in results for young children due to their movement patterns being more horizontal motion than that exhibited by older children, due to their wider base of support during gait development (Keen, 1993; Oliver *et al*., 2007). Furthermore, given the pedometer data were collected in a smaller sample (2020 cohort only), this could have reduced overall statistical power.

Both fathers and children reported significantly improved dietary behaviours. Of note, sustained improvements in healthy, nutrient-dense core foods (e.g. fruit, vegetables, water) and total dietary intake score (indicating lower intakes of energy-dense, nutrient-poor food items and higher intakes of healthy, nutrient-dense core foods) were identified. These findings are consistent with the dietary outcomes for the HYHD RCT (Ashton *et al*., 2021). The program’s emphasis on curbing coercive control (e.g. refraining from using food as a reward), enhancing meal structure (e.g. encouraging family mealtimes) and fostering autonomy (e.g. engaging children in meal preparation) could account for the favourable results. These three elements are acknowledged as the primary influences in shaping child-feeding practices (Vaughn *et al*., 2016). The identified improvements in eating habits among preschool-aged children have occurred at a crucial time because dietary habits formed early in life can influence eating habits across the lifespan (Walsh *et al*., 2016). It is also a crucial time for fathers, given that poor diet quality accounted for 15% (12.0–17.6) of all male deaths in 2019 (Murray *et al*., 2020).

At post-intervention, children significantly reduced minutes/week of screen time by 24%, while for fathers, this was slightly lower with a 14% reduction. However, this was not sustained at 12 months for either fathers or children. On average, preschool-aged children spend 113 min/day on screens (Hinkley *et al*., 2012) and therefore, a 24% reduction equates to approximately 27 min. This finding is encouraging given that this reduction is comparable to a meta-analysed mean difference (mean −17.12 min/day, 95% CI: −28.82 to −5.42) from 17 interventions (Downing *et al*., 2018), which mostly included highly controlled efficacy studies delivered by experienced health professional and/or research staff. The lack of sustained effect on screen time in both fathers and children is likely a confounding effect of the COVID-19 pandemic and subsequent strict lockdowns that occurred. Recent research has shown a vast increase in screen time usage among Australians during lockdowns, with children spending almost 27 extra hours each week on screens, and parents an extra 14.5 h each week compared with pre-lockdown (Arundell *et al*., 2021).

Notably, the improvement in dietary behaviours and reduction in screen time among fathers and children coincided with an overall improvement in fathers’ parenting practices (fathers’ self-efficacy and co-parenting scores). Specifically, the program educated fathers on using an authoritative parenting style [e.g. a combination of high parental control and positive stimuli to the child’s autonomy, including nurturing/warmth, rational communication and receptiveness (Lavrič and Naterer, 2020)] as this has been shown to improve the amount and type of limit setting, reinforcement for healthy food choices in children (Lloyd *et al*., 2015; Kiefner-Burmeister and Hinman, 2020) and lower percentages of children’s screen time (Veldhuis *et al*., 2014). In addition, the program aimed to enhance the modelling by fathers in adopting healthy eating and screen time practices at home, as positive role modelling by fathers aligns with the growing body of literature highlighting the impact of fathers on children’s lifestyle behaviours (Schoeppe *et al*., 2017; Rahill *et al*., 2020).

Overall, the positive findings and long-term impact provide encouragement for a sustained delivery model. In aligning with implementation research, there is a need to optimize the HYHD program for large-scale delivery with relatively low incremental costs (Estabrooks, 2017). Specifically, future research on the HYHD program, requires assessment of its scalability with a systematic evaluation of intervention components to identify the most efficient and effective treatment package (Hoffman *et al*., 2017). Furthermore, utilizing an appropriate implementation framework, such as the PRACTIS guide (Koorts *et al*., 2018) or the Intervention Scalability Assessment Tool (ISAT) (Milat *et al*., 2020), will be essential for mapping implementation features, engaging key stakeholders and addressing potential barriers to effective implementation and scale-up.

The current study was strengthened by a comprehensive process evaluation, long-term follow-up (12 months post-baseline), robust retention rates at 12 months (84%) and an intention-to-treat analysis. In addition, the ability to implement the program within community settings, such as schools, using trained facilitators will support future scale-up of the program. In terms of study limitations, there was an over-representation of fathers who were non-indigenous, married and with higher education levels and in full-time employment which may limit the generalisability of findings. Also, of the 2019 study cohort, 71% were part of the wait-list control group of the previous related RCT (Morgan *et al*., 2022). Importantly, these participants had not received any intervention prior to their baseline assessment in the current trial. To account for this confounder, sensitivity analyses were undertaken and there were no to very small changes observed in model estimates for fathers and children. In addition, due to the impact of COVID-19, two of the eight programs were delivered at the University of Newcastle as schools had restrictions on allowing external visitors to use the spaces. To account for this, nested random intercept models investigated the correlation between individuals by program location and facilitator. Due to the translational nature of the trial, there was no control group and self-report measures were mainly used to evaluate behaviour change. Therefore, intervention effects should be interpreted cautiously. However, it is important to note that this study was preceded by an efficacy RCT (Morgan *et al*., 2022), which had extensive measures and a comparison group and produced similar outcomes. Furthermore, validated self-report measures were chosen that we had previously used in conjunction with objective measures. Average steps/day were collected using an objective measure (YAMAX SW200 pedometer), but this was only collected in the 2020 sample only, due to high participant burden from being part of the control group in a previous related RCT. In addition, dietary outcomes were assessed using composite scores and need to be interpreted cautiously with clinical relevance taken into consideration.

## CONCLUSION

Findings demonstrate the broad replicability and adaptability of the HYHD program when delivered in the community by local trained facilitators. Notably, process results indicate that facilitators delivered the program as intended and were able to motivate and engage participants, as confirmed by the sustained high attendance and satisfaction results. This led to important improvements in health behaviours of both fathers and children at the end of the program and at 12-month follow-up. Following this, there is a need to optimize the program to be delivered at scale with relatively low incremental costs. Further investigation is needed to identify the most efficient systems, processes and contextual factors to establish how the HYHD program can be rolled out across local health districts and different community settings to achieve scale-up.

## Supplementary Material

daae095_suppl_Supplementary_Files_1

## Data Availability

The data that support the findings of this study are available upon request from the corresponding author.
