# Supplementary material for: Replicability, adaptability and long-term impact of the ‘Healthy Youngsters, Healthy Dads’ program in Newcastle, Australia
Source: Health Promot Int. 2024 Aug 12;39(4):daae095. doi: 10.1093/heapro/daae095 (PMC11317530; doi:10.1093/heapro/daae095)
Supplement: daae095_suppl_Supplementary_Files_1 [file daae095_suppl_supplementary_files_1.docx]

Supplementary File 1

**Replicability, adaptability, and long-term impact of the ‘Healthy Youngsters, Healthy Dads’ program in Newcastle, Australia.**

Lee M. Ashton^1,2^, Jacqueline A. Grounds^1,2,^, Alyce T. Barnes^4^, Emma R. Pollock^4^, Myles D Young^2,6^, Stevie-Lee Kennedy^1,2,^, Anna T. Rayward^4,5^, Daniel R. Lee^1,2^, and Philip J. Morgan^1,2,^ *.

***Corresponding Author:** Philip J Morgan, Centre for Active Living and Learning, University of Newcastle, University Drive, Awabakal Country, Callaghan, NSW 2308, Australia. Phone: +612 49 217 265. Email: philip.morgan@newcastle.edu.au.

**Table of Contents**

| **Supporting information item** | **Page** |
| --- | --- |
| **Supplementary Figure 1:** Acceptability and satisfaction findings as reported by fathers (n=121) | 2 |
| **Supplementary Figure 2:** Overall program satisfaction and facilitator rating as reported by fathers (n=121) | 3 |
| **Supplementary Table 1.** Dietary intake of fathers, stratified by timepoint. | 4 |
| **Supplementary Table 2.** Dietary intake of children, stratified by timepoint. | 7 |
| **Supplementary Table 3.** Number of observations, mean (SD) and median (Q1, Q3) for outcomes related to physical activity & parenting of dads and children. | 9 |
| **Supplementary Table 4.** Number of observations, mean (SD) and median (Q1, Q3) for average number of daily steps for dads and children. | 10 |
| **Supplementary Table 5.** Sensitivity analysis for father outcomes - comparison of model estimates, 95% CI and p-values, for models with and without a confounder for previous program participation. | 11 |
| **Supplementary Table 6.** Sensitivity analysis for child outcomes - comparison of model estimates, 95% CI and p-values, for models with and without a confounder for previous program participation. | 16 |

**Supplementary Figure 1:** Acceptability and satisfaction findings as reported by fathers (n=121)

**Supplementary figure 2:** Overall program satisfaction and facilitator rating as reported by fathers (n=121).

## Summary Tables

### Dietary Intake

Frequency tables corresponding to the dietary intake of fathers and children, including number of nights dinner was consumed in front of TV, are shown in Table 1 and Table 2, respectively.

**Table 1.** Dietary intake of fathers, stratified by timepoint.

|  | | *Time point* | | |
| --- | --- | --- | --- | --- |
| *Variable* | *Category* | *Baseline (n = 140)* | *Post Program (n = 121)* | *12-month Follow-up (n = 117)* |
| Dad usual daily serves of fruit | None | 1 (0.7%) | 1 (0.9%) | - |
|  | Less than one serve a day | 70 (50%) | 40 (35%) | 44 (39%) |
|  | 2 serves a day | 53 (38%) | 48 (42%) | 46 (41%) |
|  | 3 serves a day | 10 (7.1%) | 21 (18%) | 16 (14%) |
|  | 4 serves or more a day | 6 (4.3%) | 4 (3.5%) | 7 (6.2%) |
| Dad usual daily serves of vegetables | None | - | 1 (0.9%) | - |
|  | 1 serve or less | 24 (17%) | 8 (7.0%) | 12 (11%) |
|  | 2 serves | 63 (45%) | 41 (36%) | 35 (31%) |
|  | 3 serves | 34 (24%) | 36 (32%) | 37 (33%) |
|  | 4 serves | 11 (7.9%) | 23 (20%) | 21 (19%) |
|  | 5 serves or more | 8 (5.7%) | 5 (4.4%) | 8 (7.1%) |
| Dad frequency of hot chips/fried potato | Never or rarely | 11 (7.9%) | 13 (11%) | 12 (11%) |
|  | Less than once a week | 57 (41%) | 52 (46%) | 52 (46%) |
|  | About 1 to 2 times a week | 59 (42%) | 45 (39%) | 43 (38%) |
|  | About 3 to 4 times a week | 12 (8.6%) | 3 (2.6%) | 5 (4.4%) |
|  | About 5 to 6 times a week | - | 1 (0.9%) | 1 (0.9%) |
|  | About once a day | 1 (0.7%) | - | - |
| Dad frequency of potato crisps/salty snacks | Never or rarely | 27 (19%) | 20 (18%) | 20 (18%) |
|  | Less than once a week | 49 (35%) | 43 (38%) | 42 (37%) |
|  | About 1 to 2 times a week | 45 (32%) | 39 (34%) | 33 (29%) |
|  | About 3 to 4 times a week | 15 (11%) | 11 (9.6%) | 14 (12%) |
|  | About 5 to 6 times a week | 2 (1.4%) | - | 1 (0.9%) |
|  | About once a day | 2 (1.4%) | 1 (0.9%) | 3 (2.7%) |
| Dad frequency of confectionary consumption | Never or rarely | 17 (12%) | 17 (15%) | 14 (12%) |
|  | Less than once a week | 35 (25%) | 35 (31%) | 26 (23%) |
|  | About 1 to 2 times a week | 34 (24%) | 27 (24%) | 31 (27%) |
|  | About 3 to 4 times a week | 24 (17%) | 23 (20%) | 20 (18%) |
|  | About 5 to 6 times a week | 9 (6.4%) | 6 (5.3%) | 9 (8.0%) |
|  | About once a day | 18 (13%) | 6 (5.3%) | 12 (11%) |
|  | 2 or more times a day | 3 (2.1%) | - | 1 (0.9%) |
| Dad frequency of sweet foods consumption | Never or rarely | 26 (19%) | 26 (23%) | 21 (19%) |
|  | Less than once a week | 41 (29%) | 37 (32%) | 41 (36%) |
|  | About 1 to 2 times a week | 39 (28%) | 33 (29%) | 30 (27%) |
|  | About 3 to 4 times a week | 15 (11%) | 13 (11%) | 14 (12%) |
|  | About 5 to 6 times a week | 6 (4.3%) | 2 (1.8%) | 2 (1.8%) |
|  | About once a day | 11 (7.9%) | 2 (1.8%) | 5 (4.4%) |
|  | 2 or more times a day | 2 (1.4%) | 1 (0.9%) | - |
| Dad daily cups of water consumption | None | - | 1 (0.9%) | - |
|  | Less than one cup a day | 8 (5.7%) | - | 3 (2.7%) |
|  | About 1-2 cups a day | 14 (10%) | 5 (4.4%) | 15 (13%) |
|  | About 2-3 cups a day | 21 (15%) | 17 (15%) | 10 (8.8%) |
|  | About 3-4 cups a day | 35 (25%) | 27 (24%) | 27 (24%) |
|  | 4 cups or more a day | 62 (44%) | 64 (56%) | 58 (51%) |
| Dad daily cups of fruit juice consumption | None | 66 (47%) | 68 (60%) | 63 (56%) |
|  | Less than one cup a week | 54 (39%) | 32 (28%) | 35 (31%) |
|  | About 1-3 cups a week | 15 (11%) | 12 (11%) | 12 (11%) |
|  | About 4-6 cups a week | 2 (1.4%) | 1 (0.9%) | 2 (1.8%) |
|  | About 1-2 cups a day | 3 (2.1%) | 1 (0.9%) | 1 (0.9%) |
| Dad weekly cups of sweetened beverages | None | 39 (28%) | 38 (33%) | 39 (35%) |
|  | Less than one cup a week | 49 (35%) | 32 (28%) | 33 (29%) |
|  | About 1-3 cups a week | 27 (19%) | 28 (25%) | 25 (22%) |
|  | About 4-6 cups a week | 9 (6.4%) | 5 (4.4%) | 7 (6.2%) |
|  | About 1-2 cups a day | 9 (6.4%) | 7 (6.1%) | 5 (4.4%) |
|  | About 2-3 cups a day | 5 (3.6%) | 2 (1.8%) | 3 (2.7%) |
|  | 3 cups or more a day | 2 (1.4%) | 2 (1.8%) | 1 (0.9%) |
| Dad frequency of fast-food consumption | Never or rarely | 25 (18%) | 29 (25%) | 22 (19%) |
|  | Less than once a week | 60 (43%) | 50 (44%) | 50 (44%) |
|  | About 1 to 2 times a week | 49 (35%) | 33 (29%) | 36 (32%) |
|  | About 3 to 4 times a week | 6 (4.3%) | 2 (1.8%) | 5 (4.4%) |
| Dad days per week eating in front of TV | Never or rarely | 73 (52%) | 76 (67%) | 54 (48%) |
|  | 1 day per week | 20 (14%) | 12 (11%) | 18 (16%) |
|  | 2 days per week | 16 (11%) | 11 (9.6%) | 12 (11%) |
|  | 3 days per week | 9 (6.4%) | 5 (4.4%) | 9 (8.0%) |
|  | 4 days per week | 2 (1.4%) | 3 (2.6%) | 6 (5.3%) |
|  | 5 days per week | 8 (5.7%) | 2 (1.8%) | 6 (5.3%) |
|  | 6 days per week | 2 (1.4%) | 1 (0.9%) | 3 (2.7%) |
|  | Every night of the week | 10 (7.1%) | 4 (3.5%) | 5 (4.4%) |

**Table 2. Dietary intake of children, stratified by timepoint.**

|  | | *Time point* | | |
| --- | --- | --- | --- | --- |
| *Variable* | *Category* | *Baseline (n = 141)* | *Post Program (n = 122)* | *12-month Follow-up*  *(n = 118)* |
| Child usual daily serves of fruit | None | 1 (0.7%) | 1 (0.9%) | - |
|  | Less than one serve a day | 23 (16%) | 9 (7.8%) | 11 (9.7%) |
|  | 2 serves a day | 75 (53%) | 54 (47%) | 50 (44%) |
|  | 3 serves a day | 32 (23%) | 39 (34%) | 38 (34%) |
|  | 4 serves or more a day | 10 (7.1%) | 12 (10%) | 14 (12%) |
| Child usual daily serves of vegetable | None | 2 (1.4%) | 1 (0.9%) | - |
|  | 1 serve or less | 50 (35%) | 31 (27%) | 28 (25%) |
|  | 2 serves | 56 (40%) | 40 (35%) | 38 (34%) |
|  | 3 serves | 21 (15%) | 31 (27%) | 29 (26%) |
|  | 4 serves | 11 (7.8%) | 11 (9.6%) | 12 (11%) |
|  | 5 serves or more | 1 (0.7%) | 1 (0.9%) | 6 (5.3%) |
| Child frequency of hot chips/fried potato | Never or rarely | 28 (20%) | 21 (18%) | 17 (15%) |
|  | Less than once a week | 55 (39%) | 47 (41%) | 50 (44%) |
|  | About 1 to 2 times a week | 51 (36%) | 41 (36%) | 41 (36%) |
|  | About 3 to 4 times a week | 5 (3.5%) | 6 (5.2%) | 3 (2.7%) |
|  | About 5 to 6 times a week | 2 (1.4%) | - | 2 (1.8%) |
| Child frequency of potato crisps/salty snacks | Never or rarely | 43 (30%) | 31 (27%) | 25 (22%) |
|  | Less than once a week | 39 (28%) | 41 (36%) | 36 (32%) |
|  | About 1 to 2 times a week | 42 (30%) | 33 (29%) | 30 (27%) |
|  | About 3 to 4 times a week | 13 (9.2%) | 6 (5.2%) | 14 (12%) |
|  | About 5 to 6 times a week | 2 (1.4%) | 4 (3.5%) | 5 (4.4%) |
|  | About once a day | 2 (1.4%) | - | 3 (2.7%) |
| Child frequency of confectionary consumption | Never or rarely | 15 (11%) | 14 (12%) | 12 (11%) |
|  | Less than once a week | 33 (23%) | 37 (32%) | 36 (32%) |
|  | About 1 to 2 times a week | 55 (39%) | 38 (33%) | 38 (34%) |
|  | About 3 to 4 times a week | 25 (18%) | 19 (17%) | 21 (19%) |
|  | About 5 to 6 times a week | 5 (3.5%) | 5 (4.3%) | 1 (0.9%) |
|  | About once a day | 8 (5.7%) | 2 (1.7%) | 5 (4.4%) |
| Child frequency of sweet food consumption | Never or rarely | 7 (5.0%) | 7 (6.1%) | 7 (6.2%) |
|  | Less than once a week | 34 (24%) | 38 (33%) | 34 (30%) |
|  | About 1 to 2 times a week | 57 (40%) | 46 (40%) | 46 (41%) |
|  | About 3 to 4 times a week | 27 (19%) | 18 (16%) | 20 (18%) |
|  | About 5 to 6 times a week | 5 (3.5%) | 4 (3.5%) | 1 (0.9%) |
|  | About once a day | 11 (7.8%) | 2 (1.7%) | 5 (4.4%) |
| Child daily cups of water consumption | None | - | 2 (1.7%) | - |
|  | Less than one cup a day | 2 (1.4%) | - | - |
|  | About 1-2 cups a day | 19 (13%) | 9 (7.8%) | 9 (8.0%) |
|  | About 2-3 cups a day | 53 (38%) | 23 (20%) | 30 (27%) |
|  | About 3-4 cups a day | 43 (30%) | 44 (38%) | 49 (43%) |
|  | 4 cups or more a day | 24 (17%) | 37 (32%) | 25 (22%) |
| Child daily cups of fruit juice consumption | None | 51 (36%) | 47 (41%) | 40 (35%) |
|  | Less than one cup a week | 49 (35%) | 41 (36%) | 45 (40%) |
|  | About 1-3 cups a week | 32 (23%) | 24 (21%) | 23 (20%) |
|  | About 4-6 cups a week | 6 (4.3%) | 3 (2.6%) | 5 (4.4%) |
|  | About 1-2 cups a day | 3 (2.1%) | - | - |
| Child weekly cups of sweetened beverages | None | 111 (79%) | 93 (81%) | 86 (76%) |
|  | Less than one cup a week | 25 (18%) | 18 (16%) | 20 (18%) |
|  | About 1-3 cups a week | 5 (3.5%) | 4 (3.5%) | 5 (4.4%) |
|  | About 1-2 cups a day | - | - | 2 (1.8%) |
| Child frequency of fast-food consumption | Never or rarely | 40 (28%) | 35 (30%) | 33 (29%) |
|  | Less than once a week | 61 (43%) | 49 (43%) | 50 (44%) |
|  | About 1 to 2 times a week | 38 (27%) | 28 (24%) | 28 (25%) |
|  | About 3 to 4 times a week | 2 (1.4%) | 3 (2.6%) | 2 (1.8%) |
| Child days per week eating in front of TV | Never or rarely | 71 (50%) | 76 (66%) | 53 (47%) |
|  | 1 day per week | 30 (21%) | 16 (14%) | 24 (21%) |
|  | 2 days per week | 8 (5.7%) | 9 (7.8%) | 16 (14%) |
|  | 3 days per week | 10 (7.1%) | 3 (2.6%) | 7 (6.2%) |
|  | 4 days per week | 4 (2.8%) | 6 (5.2%) | 4 (3.5%) |
|  | 5 days per week | 7 (5.0%) | 1 (0.9%) | 3 (2.7%) |
|  | 6 days per week | 4 (2.8%) | 2 (1.7%) | 1 (0.9%) |
|  | Every night of the week | 7 (5.0%) | 2 (1.7%) | 5 (4.4%) |

### Physical activity and parenting outcomes

Summary tables corresponding to the physical activity, screentime, and parenting outcomes of dads and children, stratified by timepoint, are shown in Table 3. One participant reported a value of 6790 minutes for Dad weekly minutes of moderate-vigorous physical activity (PA) at the post program timepoint with this value considered to be an entry error and set to missing for both the summary calculations and regression models.

**Table 3.** Number of observations, mean (SD) and median (Q1, Q3) for outcomes related to physical activity & parenting of dads and children.

|  | | *Time point* | | |
| --- | --- | --- | --- | --- |
| *Variable* | *Category* | *Baseline*  *(n = 140 fathers n= 141 children)* | *Post Program*  *(n = 121 fathers n= 122 children)* | *12-months*  *(n = 117 fathers n= 118 children)* |
| Dad co-physical activity with child & family- days/week | n | 140 | 118 | 117 |
|  | mean (SD) | 2 (2) | 3 (2) | 3 (2) |
|  | median  (Q1, Q3) | 2  (1, 3) | 3  (2, 4) | 3  (2, 4) |
| Dad co-physical activity with child only- days/week | n | 140 | 118 | 117 |
|  | mean (SD) | 2 (1) | 2 (2) | 2 (1) |
|  | median  (Q1, Q3) | 1  (0, 3) | 2  (1, 3) | 2  (1, 3) |
| Dad weekly minutes of moderate-vigorous PA | n | 135 | 116 | 100 |
|  | mean (SD) | 158.5 (133.7) | 186.4 (120.7) | 200.5 (157.3) |
|  | Median  (Q1, Q3) | 140.0  (60.0, 210.0) | 150.0  (97.5, 255.0) | 180.0  (102.5, 262.5) |
| Dad weekly average minutes/day screentime | n | 140 | 115 | 116 |
|  | mean (SD) | 110.5 (62.2) | 92.2 (52.3) | 101.4 (53.2) |
|  | median  (Q1, Q3) | 111.4 (62.1, 145.7) | 85.7  (51.4, 120.0) | 94.3  (60.0, 129.3) |
| Child weekly average minutes/day screentime | n | 140 | 115 | 114 |
|  | mean (SD) | 89.6 (52.7) | 67.2 (39.7) | 92.7 (50.3) |
|  | median  (Q1, Q3) | 77.1  (51.4, 120.0) | 60.0  (35.7, 81.4) | 79.3  (57.9, 120.0) |
| Fathering Self Efficacy scale score | n | 140 | 115 | 113 |
|  | mean (SD) | 114 (14) | 120 (12) | 118 (17) |
|  | Median  (Q1, Q3) | 116  (107, 123) | 120  (113, 127) | 117  (111, 127) |
| Feinberg co-parenting scale – Brief measure score | n | 134 | 111 | 98 |
|  | mean (SD) | 51 (7) | 52 (7) | 52 (6) |
|  | median  (Q1, Q3) | 52  (47, 56) | 53  (48, 57) | 52  (49, 56) |

Pedometry data obtained from fathers and children participating in the 2020 study cohort, stratified by timepoint, is summarised in Table 4

**Table 4.** Number of observations, mean (SD) and median (Q1, Q3) for average number of daily steps for dads and children.

|  | | *Time point* | |
| --- | --- | --- | --- |
| *Variable* | *Category* | *Baseline* | *Post Program* |
| Dad Average Steps | n | 75 | 62 |
|  | mean (SD) | 8378 (2794) | 8320 (2697) |
|  | median (Q1, Q3) | 7907 (6417, 9806) | 8262 (6319, 10889) |
| Child Average Steps | n | 74 | 62 |
|  | mean (SD) | 9423 (3020) | 9236 (2629) |
|  | median (Q1, Q3) | 9000 (7402, 11007) | 9135 (7549, 10958) |

## Sensitivity analysis

The sensitivity of the mixed effects regression models to the presence of a confounder for previous involvement to the program was examined. Previous involvement to the program only impacted the 2019 study cohort.

A comparison of model estimates, 95% CI and p-values from models with (Main Analysis) and without (Sensitivity Analysis) the confounder for previous participation is shown in Table 5 for father outcomes and in Table 6 for child outcomes.

Table 5. Sensitivity analysis for father outcomes - comparison of model estimates, 95% CI and p-values, for models with and without a confounder for previous program participation.

|  | | | | | | *Main Analysis* | | *Sensitivity Analysis* | |
| --- | --- | --- | --- | --- | --- | --- | --- | --- | --- |
| *Program Outcome* | *Model Distribution* | *Type of Estimate* | *Number of Obs Used* | *Predictor* | *Comparison* | *Estimate*  *(95% CI)* | *P-value* | *Estimate*  *(95% CI)* | *P-value* |
| Co-parenting score | Gaussian | Average change | 343 | Timepoint | Post program vs Baseline | 1.19  (0.20, 2.18) | 0.02 | 1.20  (0.21, 2.19) | 0.02 |
|  |  |  | . | Timepoint | 12-month follow-up vs Baseline | 1.41  (0.37, 2.44) | 0.01 | 1.41  (0.38, 2.45) | 0.01 |
|  |  |  | . | Previous involvement in RCT | Involved vs not involved | -1.85  (-5.22, 1.51) | 0.28 |  | . |
|  |  |  | . | Cohort year | 2020 vs 2019 | -0.35  (-3.53, 2.83) | 0.83 | 0.98  (-1.09, 3.05) | 0.35 |
| Co-physical activity - family | Binary | Odds ratio | 375 | Time-point | Post program vs Baseline | 4.29  (1.43, 12.89) | 0.01 | 4.23  (1.41, 12.67) | 0.01 |
|  |  |  | . | Time-point | 12-month follow-up vs Baseline | 12.68  (2.79, 57.62) | 0.00 | 12.53  (2.75, 56.99) | 0.00 |
|  |  |  | . | Previous involvement in RCT | Involved vs not involved | 2.40  (0.44, 13.13) | 0.31 |  | . |
|  |  |  | . | Cohort year | 2020 vs 2019 | 3.00  (0.61, 14.78) | 0.18 | 1.66  (0.53, 5.16) | 0.38 |
|  | Gamma | Rate ratio | 343 | Time-point | Post program vs Baseline | 1.27  (1.13, 1.44) | 0.00 | 1.27  (1.13, 1.44) | 0.00 |
|  |  |  | . | Time-point | 12-month follow-up vs Baseline | 1.11  (0.98, 1.25) | 0.10 | 1.10  (0.98, 1.25) | 0.10 |
|  |  |  | . | Previous involvement in RCT | Involved vs not involved | 1.05  (0.86, 1.30) | 0.61 |  | . |
|  |  |  | . | Cohort year | 2020 vs 2019 | 1.03  (0.85, 1.26) | 0.74 | 0.99  (0.88, 1.13) | 0.94 |
| Co-physical activity - solo | Binary | Odds ratio | 375 | Time-point | Post program vs Baseline | 8.74  (3.09, 24.69) | 0.00 | 8.73  (3.09, 24.67) | 0.00 |
|  |  |  | . | Time-point | 12-month follow-up vs Baseline | 4.07  (1.69, 9.82) | 0.00 | 4.08  (1.69, 9.84) | 0.00 |
|  |  |  | . | Previous involvement in RCT | Involved vs not involved | 0.44  (0.07, 2.89) | 0.39 |  | . |
|  |  |  | . | Cohort year | 2020 vs 2019 | 0.45  (0.08, 2.71) | 0.39 | 0.83  (0.28, 2.45) | 0.74 |
|  | Gamma | Rate ratio | 309 | Time-point | Post program vs Baseline | 1.27  (1.12, 1.43) | 0.00 | 1.27  (1.12, 1.43) | 0.00 |
|  |  |  | . | Time-point | 12-month follow-up vs Baseline | 1.05  (0.92, 1.18) | 0.48 | 1.05  (0.92, 1.18) | 0.48 |
|  |  |  | . | Previous involvement in RCT | Involved vs not involved | 1.07  (0.82, 1.39) | 0.63 |  | . |
|  |  |  | . | Cohort year | 2020 vs 2019 | 0.96  (0.75, 1.23) | 0.76 | 0.92  (0.78, 1.09) | 0.33 |
| Energy-dense, nutrient poor foods | Gaussian | Average change | 367 | Timepoint | Post program vs Baseline | 1.61  (1.03, 2.19) | 0.00 | 1.61  (1.03, 2.20) | 0.00 |
|  |  |  | . | Timepoint | 12-month follow-up vs Baseline | 0.84  (0.26, 1.43) | 0.00 | 0.85  (0.26, 1.43) | 0.00 |
|  |  |  | . | Previous involvement in RCT | Involved vs not involved | -1.97  (-4.33, 0.40) | 0.10 |  | . |
|  |  |  | . | Cohort year | 2020 vs 2019 | -2.16  (-4.40, 0.08) | 0.06 | -0.74  (-2.20, 0.72) | 0.32 |
| Father self-efficacy | Gaussian | Average change | 368 | Timepoint | Post program vs Baseline | 5.66  (3.05, 8.27) | 0.00 | 5.67  (3.07, 8.28) | 0.00 |
|  |  |  | . | Timepoint | 12-month follow-up vs Baseline | 3.52  (0.90, 6.14) | 0.01 | 3.53  (0.91, 6.15) | 0.01 |
|  |  |  | . | Previous involvement in RCT | Involved vs not involved | -2.11  (-8.44, 4.22) | 0.51 |  | . |
|  |  |  | . | Cohort year | 2020 vs 2019 | 3.56  (-2.42, 9.53) | 0.24 | 5.07  (1.18, 8.95) | 0.01 |
| Minutes of physical activity | Negative binomial | Rate ratio | 351 | Timepoint | Post program vs Baseline | 1.30  (1.14, 1.49) | 0.00 | 1.30  (1.14, 1.48) | 0.00 |
|  |  |  | . | Timepoint | 12-month Follow-up vs Baseline | 1.37  (1.19, 1.57) | 0.00 | 1.37  (1.19, 1.57) | 0.00 |
|  |  |  | . | Previous involvement in RCT | Involved vs not involved | 1.18  (0.76, 1.83) | 0.46 |  | . |
|  |  |  | . | Cohort year | 2020 vs 2019 | 1.45  (0.95, 2.19) | 0.08 | 1.29 (0.98, 1.69) | 0.07 |
| Nights dinner in front of TV | Binary | Odds ratio | 367 | Time-point | Post program vs Baseline | 3.82  (1.61, 9.06) | 0.00 | 3.84  (1.61, 9.11) | 0.00 |
|  |  |  | . | Time-point | 12-month follow-up vs Baseline | 0.66  (0.30, 1.45) | 0.30 | 0.66  (0.30, 1.46) | 0.30 |
|  |  |  | . | Previous involvement in RCT | Involved vs not involved | 0.41  (0.04, 3.72) | 0.43 |  | . |
|  |  |  | . | Cohort year | 2020 vs 2019 | 2.43  (0.29, 20.12) | 0.41 | 4.68  (1.13, 19.37) | 0.03 |
|  | Gamma | Rate ratio | 164 | Time-point | Post program vs Baseline | 0.78  (0.65, 0.94) | 0.01 | 0.78  (0.65, 0.94) | 0.01 |
|  |  |  | . | Time-point | 12-month follow-up vs Baseline | 1.00  (0.85, 1.18) | 0.98 | 1.00  (0.85, 1.18) | 0.98 |
|  |  |  | . | Previous involvement in RCT | Involved vs not involved | 1.12  (0.70, 1.79) | 0.62 |  | . |
|  |  |  | . | Cohort year | 2020 vs 2019 | 1.08  (0.68, 1.70) | 0.75 | 0.99  (0.74, 1.33) | 0.95 |
| Healthy, nutrient-dense core foods | Gaussian | Average change | 367 | Timepoint | Post program vs Baseline | 0.99  (0.67, 1.31) | 0.00 | 0.99  (0.67, 1.31) | 0.00 |
|  |  |  | . | Timepoint | 12-month follow-up vs Baseline | 0.81  (0.49, 1.13) | 0.00 | 0.81  (0.49, 1.13) | 0.00 |
|  |  |  | . | Previous involvement in RCT | Involved vs not involved | -0.95  (-1.96, 0.07) | 0.07 |  | . |
|  |  |  | . | Cohort year | 2020 vs 2019 | -0.63  (-1.60, 0.34) | 0.20 | 0.06  (-0.61, 0.73) | 0.86 |
| Total dietary intake | Gaussian | Average change | 367 | Timepoint | Post program vs Baseline | 2.60  (1.93, 3.28) | 0.00 | 2.61  (1.93, 3.28) | 0.00 |
|  |  |  | . | Timepoint | 12-month follow-up vs Baseline | 1.66  (0.98, 2.34) | 0.00 | 1.66  (0.99, 2.34) | 0.00 |
|  |  |  | . | Previous involvement in RCT | Involved vs not involved | -2.90  (-5.71, -0.09) | 0.04 |  | . |
|  |  |  | . | Cohort year | 2020 vs 2019 | -2.78  (-5.44, -0.12) | 0.04 | -0.69  (-2.43, 1.05) | 0.44 |
| Weekly average minutes of screentime | Negative binomial | Rate ratio | 371 | Timepoint | Post program vs Baseline | 0.86  (0.78, 0.94) | 0.00 | 0.86  (0.78, 0.94) | 0.00 |
|  |  |  | . | Timepoint | 12-month follow-up vs Baseline | 0.93  (0.85, 1.01) | 0.10 | 0.93  (0.85, 1.01) | 0.10 |
|  |  |  | . | Previous involvement in RCT | Involved vs not involved | 1.11  (0.82, 1.52) | 0.49 |  | . |
|  |  |  | . | Cohort year | 2020 vs 2019 | 1.12  (0.83, 1.50) | 0.47 | 1.03  (0.84, 1.26) | 0.77 |

Table 6. Sensitivity analysis for Child outcomes - comparison of model estimates, 95% CI and p-values, for models with and without a confounder for previous program participation.

|  | | | | | | *Main Analysis* | | *Sensitivity Analysis* | |
| --- | --- | --- | --- | --- | --- | --- | --- | --- | --- |
| *Program Outcome* | *Model Distribution* | *Type of Estimate* | *Number of Obs Used* | *Predictor* | *Comparison* | *Estimate (95% CI)* | *P-value* | *Estimate*  *(95% CI)* | *P-value* |
| Energy-dense, nutrient poor foods | Gaussian | Average change | 369 | Timepoint | Post program vs Baseline | 0.84  (0.32, 1.35) | 0.00 | 0.84  (0.33, 1.35) | 0.00 |
|  |  |  | . | Timepoint | 12-month follow-up vs Baseline | 0.03  (-0.49, 0.54) | 0.92 | 0.03  (-0.49, 0.55) | 0.91 |
|  |  |  | . | Previous involvement in RCT | Involved vs not involved | -1.14  (-3.25, 0.97) | 0.29 |  | . |
|  |  |  | . | Cohort year | 2020 vs 2019 | -1.13  (‑3.12, 0.86) | 0.27 | -0.31  (-1.59, 0.98) | 0.64 |
| Nights dinner in front of TV | Binary | Odds ratio | 369 | Time-point | Post program vs Baseline | 3.76  (1.68, 8.41) | 0.00 | 3.74  (1.67, 8.37) | 0.00 |
|  |  |  | . | Time-point | 12-month follow-up vs Baseline | 0.73  (0.35, 1.55) | 0.41 | 0.73  (0.35, 1.54) | 0.41 |
|  |  |  | . | Previous involvement in RCT | Involved vs not involved | 1.89  (0.25, 14.06) | 0.53 |  | . |
|  |  |  | . | Cohort year | 2020 vs 2019 | 1.06  (0.16, 6.94) | 0.95 | 0.67  (0.20, 2.26) | 0.51 |
|  | Gamma | Rate ratio | 169 | Time-point | Post program vs Baseline | 0.77  (0.63, 0.94) | 0.01 | 0.77  (0.63, 0.94) | 0.01 |
|  |  |  | . | Time-point | 12-month follow-up vs Baseline | 0.96  (0.80, 1.15) | 0.68 | 0.97  (0.81, 1.16) | 0.72 |
|  |  |  | . | Previous involvement in RCT | Involved vs not involved | 1.10  (0.68, 1.78) | 0.69 |  | . |
|  |  |  | . | Cohort year | 2020 vs 2019 | 1.19  (0.76, 1.86) | 0.45 | 1.11  (0.83, 1.49) | 0.49 |
| Healthy, nutrient-dense core foods | Gaussian | Average change | 369 | Timepoint | Post program vs Baseline | 0.97  (0.65, 1.28) | 0.00 | 0.96  (0.65, 1.28) | 0.00 |
|  |  |  | . | Timepoint | 12-month follow-up vs Baseline | 1.01  (0.69, 1.33) | 0.00 | 1.01  (0.69, 1.32) | 0.00 |
|  |  |  | . | Previous involvement in RCT | Involved vs not involved | 1.12  (0.17, 2.07) | 0.02 |  | . |
|  |  |  | . | Cohort year | 2020 vs 2019 | 0.62  (-0.28, 1.51) | 0.18 | -0.19  (-0.78, 0.40) | 0.53 |
| Total dietary intake | Gaussian | Average change | 369 | Timepoint | Post program vs Baseline | 1.81  (1.22, 2.39) | 0.00 | 1.81  (1.22, 2.39) | 0.00 |
|  |  |  | . | Timepoint | 12-month follow-up vs Baseline | 1.03  (0.43, 1.62) | 0.00 | 1.03  (0.43, 1.62) | 0.00 |
|  |  |  | . | Previous involvement in RCT | Involved vs not involved | -0.03  (-2.50, 2.44) | 0.98 |  | . |
|  |  |  | . | Cohort year | 2020 vs 2019 | -0.50  (-2.83, 1.83) | 0.67 | -0.48  (-1.98, 1.02) | 0.53 |
| Weekly average minutes of screentime | Negative binomial | Rate ratio | 372 | Timepoint | Post program vs Baseline | 0.76  (0.70, 0.82) | 0.00 | 0.76  (0.70, 0.82) | 0.00 |
|  |  |  | . | Timepoint | 12-month follow-up vs Baseline | 1.03  (0.95, 1.12) | 0.47 | 1.03  (0.95, 1.12) | 0.47 |
|  |  |  | . | Previous involvement in RCT | Involved vs not involved | 1.16  (0.85, 1.58) | 0.36 |  | . |
|  |  |  | . | Cohort year | 2020 vs 2019 | 1.21  (0.90, 1.62) | 0.21 | 1.09  (0.90, 1.32) | 0.40 |
